# Evaluation of the role of *Spirulina Platensis* in diets containing flaxseed oil on performance, antioxidant status, ovarian follicles, egg shelf life, and yolk fatty acid profile in laying hens

**DOI:** 10.1016/j.vas.2025.100544

**Published:** 2025-11-16

**Authors:** Javad Ghanouni-Bostanabad, Seyyed Ali Mirghelenj, Mohsen Daneshyar, Ali Hashemi, Sina Payvastegan, Amir Mosayyeb Zadeh, Hamzeh Ghaderi-Chaparabad, Motaleb Ebrahimi

**Affiliations:** Department of Animal Science, College of Agriculture and Natural Resources, Urmia University, Urmia 5715193444, Iran

**Keywords:** Spirulina platensis, Flaxseed oil, Follicles, Fatty acids, Egg storage

## Abstract

The present study was conducted to investigate the effect of *Spirulina Platensis* (**SP**) supplementation in diets containing different levels of flaxseed oil on production performance, antioxidant status, ovarian characteristics, egg quality during storage and yolk fatty acid profile in laying hens. For this purpose, 288 laying hens (Hy-line-W36, 70 weeks of age) were used in a completely randomized design with a 2 × 3 factorial arrangement with 6 replications and 8 birds per replication. The experimental treatments included diets containing 3 levels of flaxseed oil (0, 1.5 and 3 % of the diet) and 2 levels of the SP (0 and 5 g/kg of the diet). The results showed that the use of 5 g/kg of Spirulina in flaxseed oil diet at levels of 1.5 and 3 % significantly improved the laying percentage, egg weight and feed conversion ratio (*P* < 0.05). Consumption of flaxseed oil without SP caused a decrease in total antioxidant capacity and an increase in malondialdehyde (**MDA**) in serum and liver, while the addition of 5 g/kg of SP compensated and improved these effects (*P* < 0.05). The interactive effects indicated that SP supplementation in 1.5 and 3 % flaxseed oil-containing diets had a synergistic effect on large yellow follicles weight (*P* < 0.05). The three-way interaction (flaxseed oil × SP × storage) revealed that SP supplementation significantly improved Haugh unit values during storage compared to treatments without SP (*P* < 0.05). In addition, increasing the level of flaxseed oil increased omega-3 unsaturated fatty acids including alpha-linolenic acid, eicosapentaenoic acid, and docosahexaenoic acid (**DHA**) and decreased the omega-6/ omega-3 ratio in egg yolk, and simultaneous consumption of SP showed more increasing effects on the concentration of omega-3 fatty acids (*P* < 0.05). Also, a linear and positive relationship was observed between the concentration of DHA in egg yolk and the amount of MDA in liver tissue (r = 0.978, r² = 0.957), indicating a very strong correlation between egg enrichment with omega-3 fatty acids and increased lipid peroxidation in the liver. While a conventional egg supplied only one-twentieth and one-sixteenth of the daily omega-3 requirement for men and women, respectively, a single enriched egg with 3 % flaxseed oil plus SP provided approximately one-eighth of the daily requirement for men and one-seventh for women. In general, the addition of flaxseed oil to the diet of laying hens, while improving production performance and yolk fatty acid profile, increased lipid peroxidation and decreased egg stability during storage. However, simultaneous supplementation with SP not only increased production performance and ovarian follicles, but also can be an effective strategy to produce functional eggs with enhanced health benefits, improved oxidative stability, and extended shelf life.

## 1 Introduction

Omega-3 fatty acids play an important role in human nutrition due to their role in reducing the incidence of cardiovascular and inflammatory diseases (Sherratt et al., 2023). These diseases are an increasing problem due to the consumption of animal fats and partially hydrogenated vegetable oils. Enriching eggs with omega-3 fatty acids is an effective strategy to ensure an adequate supply of these fatty acids in the community. The production of such eggs is possible by adding common sources of unsaturated fatty acids (**UFAs**) (such as flaxseed oil, fish oil or seaweed, etc.) to the diet of laying hens (Elkin & Harvatine, 2023). Flaxseed oil is included in poultry diets with the aim of utilizing the energy-generating capacity and improving the content of omega-3 fatty acids in the eggs and meat and thus providing as enriched food to consumers (Gao et al., 2021). Flaxseed oil, one of the richest plant-based sources of omega-3, contains high concentrations of α-linolenic acid (C18:3 n-3), a metabolic precursor to long-chain omega-3 fatty acids such as eicosapentaenoic acid (**EPA**) and docosahexaenoic acid (**DHA**). These fatty acids are essential for cardiovascular health, anti-inflammatory regulation, and neural development (Lee et al., 2021). Dietary inclusion of flaxseed oil in laying hen rations has been shown to significantly increase the concentration of α-linolenic acid and other omega-3 fatty acids in egg yolk without negatively affecting egg production or quality (Li et al., 2023). In another study, Saleh (2013) reported that the use of omega-3 fatty acid sources (3.5 % fish oil) in the diet of laying hens increased body weight gain and egg quality, while yolk cholesterol decreased by 14.5 %.

In addition to the positive effects of these enriched eggs on human health, it has been reported that omega-3 fatty acids from flaxseed oil have positive effects on ovarian function and follicle number (Chu et al., 2024). Several studies have investigated the influence of dietary lipids on ovarian follicular development in various species. Ebeid et al. (2008) evaluated the effects of different levels of dietary fish oil supplementation on follicular development in hens without the inclusion of dietary antioxidants. It has been reported that the addition of 1 g/kg flaxseed to the diet of older laying hens had a positive effect on plasma luteinizing hormone, follicle-stimulating hormone, estradiol-17β and ultimately on laying performance (Saleh et al., 2019). Likewise, research by Leroy et al. (2014) suggested that the number of ovarian follicles be increased through dietary polyunsaturated fatty acids (**PUFAs**), although the role of antioxidants remained unexamined. Similarly, Mahla et al. (2017) reported enhanced preovulatory follicle numbers and ovulation rates in goats supplemented with flaxseed oil; however, the potential influence of antioxidants was not considered. It has been hypothesized that specific fatty acids, notably EPA and DHA, provided by flaxseed oil, contribute significantly to reproductive performance (Fouladi-Nashta et al., 2009). Nevertheless, while PUFA-induced changes may affect ovarian dynamics and oocyte characteristics, the issue of lipid peroxidation has often been overlooked. It has been reported that products containing higher proportions of omega-3 fatty acids make them more susceptible to lipid oxidation, which is an undesirable characteristic (Musati et al., 2025).

Given that antioxidants can counteract the harmful effects of reactive oxygen species on reproductive organs and products such as eggs, they are regarded as a crucial component in avian nutrition. Therefore, the use of antioxidants in flaxseed oil-containing diets seems to be crucial. *Spirulina Platensis* (**SP**) is a blue-green algae (cyanobacteria) known for its high concentration of protein, essential amino acids, vitamins, minerals, and bioactive substances (Anvar & Nowruzi, 2021). Beyond these essential nutrients, SP contains an antioxidant compound called phycocyanin. Phycocyanin, a pigment-protein complex found in SP, has potent antioxidant properties and helps neutralize free radicals, thereby protecting cells from oxidative damage (Fernandes et al., 2023). It has been reported that SP supplementation at levels of 5 and 10 g/kg increased serum glutathione peroxidase and superoxide dismutase activity, while malondialdehyde decreased (Abdel-Moneim et al., 2022). It has also been reported that adding SP to the diet has the potential to improve performance, egg quality, reduce serum cholesterol, and increase the antioxidant capacity of egg yolk (Vui et al., 2024). The internal quality of eggs decreases significantly during storage (Mirghelenj et al., 2017). Therefore, it is likely that the use of antioxidant sources can help neutralize free radicals, protect cells from oxidative damage, and ultimately increase egg shelf life (Ebrahimi et al., 2025). Antioxidant sources can also prevent further oxidation of fatty acids in eggs (Rouhanipour et al., 2022).

According to the information provided, it can be stated that the use of flaxseed oil in the diet of laying hens as a source of long-chain omega-3 fatty acids is of great importance for the production of omega-3 eggs and maintaining the health of the community. On the other hand, due to the high content of UFAs in flaxseed oil, there is a possibility of oxidation of egg lipids even during storage, which causes the production of harmful compounds and free radicals. In these circumstances, the use of natural antioxidants such as the microalgae SP along with flaxseed oil is of particular importance. To our knowledge, no previous study has simultaneously evaluated the effects of SP and flaxseed oil on production traits, ovarian follicle dynamics, egg shelf life, and yolk fatty acid content in aged laying hens. Therefore, the present study was conducted to investigate the effect of SP supplementation in diets containing different levels of flaxseed oil on the performance, antioxidant parameters, follicle growth, egg quality during storage and yolk fatty acid profile in aged laying hens.

## 2 Materials and methods

### 2.1 Animal ethics

This study was approved by the Animal Care Committee and Animal Research Ethics Board of Urmia University, Urmia, Iran (Approval No:IR-UU-AEC-3/116).

### 2.2 Birds and experimental treatments

In this study, 288 laying hens (Hy-line-W36, 70 weeks of age) were used. Birds with similar body weight (1530 ± 50 g) with an average egg production of 75 % were selected and allocated in a completely randomized design with a 2 × 3 factorial arrangement with 6 replications and 8 birds per replication. In order to adapt to the experimental conditions, the birds were first fed with the basal diet for 2 weeks and then with the experimental treatments for 8 weeks (56 days). The experimental treatments included diets containing 3 levels of flaxseed oil (0, 1.5 and 3 % of the diet) and 2 levels of SP (0 and 5 g/kg of diet), which were formulated according to the nutritional recommendations of the Hy-line-W36 strain in 2020 (Table 1). The fatty acids profile of the diets used in the present experiment is shown in Table 1. The fatty acid profile of the basal diet and the flaxseed oil used in the experiment was determined by gas chromatography (Agilent 6890, Agilent CO, USA). Based on these results, the fatty acid composition of the diets containing 1.5 % and 3 % flaxseed oil was calculated by combining the analyzed values of the basal diet with the corresponding fatty acid composition of flaxseed oil according to their inclusion levels. The light schedule was 16 h of light with an intensity of 30 lx and 8 h of darkness. During the experiment, the temperature and humidity of the room were maintained at 20 to 25 °C and 40 to 50 %, respectively.

**Table 1:** The ingredient and chemical composition of experimental diets.

Ingredient (% of diet)	Control diet	1.5 % Flaxseed oil	3 % Flaxseed oil
Corn	60.44	57.25	53.06
Soybean meal (44 %)	25.36	25.23	26.14
Wheat bran	-	1.00	2.00
Soybean oil	0.3	0.3	0.3
Flaxseed oil	-	1.50	3.00
Dicalcium phosphate	2.16	2.27	2.48
Calcium carbonate	10.63	11.33	11.90
Salt	0.32	0.32	0.32
Sodium bicarbonate	0.10	0.10	0.10
DL- Methionine	0.09	0.10	0.10
Vitamin premix	0.30	0.30	0.30
Mineral premix	0.30	0.30	0.30
Chemical composition			
Metabolisable energy (kcal/kg)	2680	2680	2680
Crude protein (%)	15.50	15.50	15.50
Calcium (%)	4.75	4.75	4.75
Available phosphorus (%)	0.39	0.39	0.39
Sodium (%)	0.17	0.17	0.17
Methionine (%)	0.36	0.36	0.36
Methionine + Cysteine (%)	0.64	0.64	0.64
Lysine (%)	0.79	0.79	0.79
Fatty acid (%)			
C14:0	0.11	0.29	0.48
C16:0	26.84	29.72	32.05
C16:1	0.068	0.047	0.045
C18:0	4.02	4.51	5.87
C18:1 n-9	30.84	28.54	25.40
C18:1 n-7	0.74	0.67	0.63
C18:2 n-6	31.62	28.18	24.89
C18:3 n-3	0.26	0.29	0.48
C20:4 n-6	0.28	0.35	0.47
C20:5 n-3	0.03	0.89	2.21
C22:6 n-3	0.02	1.03	1.50
SFA	27.85	31.46	36.16
PUFA	32.27	30.52	29.31
PUFA: SFA	1.15	0.97	0.81
ω-3	0.21	2.11	3.89
ω-6	32.41	28.40	25.74
ω-6: ω-3	154.33	13.45	6.61
LA: ALA	112.9	93.93	47.32

^1^Supplied per kilogram of diet: Vitamin A, IU10000; Vitamin 3D, IU2500; Vitamin E, IU10; Vitamin B_1_, 22 mg; Vitamin B_2_, 4 mg; Vitamin B_3_, 8 mg; Vitamin B_4_, 2 mg; Vitamin B_9_, 0.56 mg; Vitamin B_12_, 0.015 mg; Choline, 200 mg.

^2^Supplied per kilogram of diet: Manganese, 80 mg; Iron, 50 mg; Zinc, 60 mg; Copper, 12 mg; Sodium Selenite, 0.3 mg.

PUFA: polyunsaturated fatty acids, SFA: saturated fatty acids, LA: linoleic acid, ALA: a-linolenic acid

### 2.3 Chemical analysis and antioxidant activity of spirulina platensis (invitro assay)

SP powder used in this experiment was obtained from Algae Pars Company, Shiraz, Iran. The amounts of dry matter (using an oven), ash (using a furnace), crude protein (using the Kjeldahl method), ether extract (using Soxhlet extraction), gross energy (using a bomb calorimeter: Parr 6200 bomb calorimeter, Parr Instruments Co., Moline, IL), calcium and phosphorus were measured in the laboratory (AOAC, 2006). The profile of some essential amino acids was extracted by the company itself using HPLC. Radical capturing activity of the SP extract was evaluated by 1,1-diphenyl-2-picrylhydrazyl (DPPH assay) according to Mtaki et al. (2020) procedure. Briefly, a series of extract concentrations with different ratios of extracts to methanol 80 % were prepared as 1: 0.1, 1:0.01, 1:0.001, 1:0.0001 and 1:0.00001. About 4.9 mL of SP extract added to 0.1 mL of 5 mM DPPH radical in methanol 80 %, vortexed in dark room for 30 min. Then, the absorbance of SP extract containing DPPH (A_1_), without DPPH (A_s_), DPPH solution without SP (A_o_, called control) were assessed at 517 nm by using spectrophotometer (Dynamico- HALO DB-20 UV Vision Double Beam). Radical scavenging activity of each sample was calculated by the following formula: DPPH radical scavenging activity (%) = [A0−(A1−As) A0] × 100.

The amounts of dry matter were 88 %, ash 9 %, crude protein 55 %, ether extract 4.5 %. The values ​​of gross energy were 4300 kcal/kg, metabolizable energy 2580 kcal/kg, calcium 0.25 % and phosphorus 1.1 %. Also, the DPPH content in SP was 65 %.

### 2.4 Performance

To evaluate performance indicators, eggs were collected twice a day (10 am and 4 pm) and the total number and weight of eggs were recorded at the end of each day. Egg mass, feed intake and feed conversion ratio (**FCR**) were calculated weekly. Thus, the percentage of egg production was calculated by dividing the number of eggs produced in each replicate by the hen's day. Egg mass was obtained by multiplying the average egg weight of each replicate by the percentage of egg production. Also, FCR was obtained by dividing feed intake by egg mass. The following equation was used to determine the feed cost per kilogram of eggs produced (Nampijja et al., 2023).

Feedcostperkilogramofeggs=feedconversionratio×priceperkilogramoffeedconsumed

### 2.5 Assessment of antioxidant parameters

To evaluate antioxidant parameters, two serum samples were collected from each replicate (12 samples per treatment) at the end of the experimental period. Blood samples were collected from the wing vein after 4 h of feed withdrawal, and the blood-filled 5 mL syringes were placed at an inclined position at room temperature for 4 h to allow serum separation. To obtain clearer and higher-quality serum samples, they were centrifuged at 3000 × g for 15 min at 4 °C. The serum samples were then transferred at –20 °C to the Biochemistry Laboratory. For the assessment of liver antioxidant status, six birds from each treatment were slaughtered at the end of the experiment. After opening the carcasses, liver samples were collected and sent to the laboratory for the evaluation of total antioxidant capacity (**TAC**), malondialdehyde (**MDA**) activity, and the activities of glutathione peroxidase (**GSH-Px**) and superoxide dismutase (**SOD**) enzymes. Both liver and serum samples were analyzed using an automated analyzer (Autoanalyzer; Technicon RA 1000, Bayer) and diagnostic kits manufactured by Pars Azmoon Co. The antioxidant parameters in serum (TAC and MDA activity) and liver (TAC, MDA activity, GSH-Px, and SOD) were measured according to the protocols recommended by the kit manufacturer (Pars Azmoon).

### 2.6 Ovarian follicle assessment

At the end of the experiment, two birds from each replicate were selected (Similar weight) and then killed. Follicles were subsequently counted and weighed following the procedure outlined by Ebrahimi et al. (2025). Follicles were classified into four groups based on their diameter: large yellow follicles (**LYF**; >10 mm), small yellow follicles (**SYF**; 5–10 mm), large white follicles (**LWF**; 3–5 mm), and medium white follicles (**MWF**; 1–3 mm). Yellow follicles under 5 mm were categorized as atretic follicles (Renema et al., 1995). To measure the weight of follicles, first the total weight of stroma including all follicles on it was recorded. Then the weight of stroma was measured after removing LYF and SYF follicles. Finally, the weight of the largest follicle ready for ovulation (**F1**) was measured (Ebrahimi et al., 2025).

### 2.7 Egg quality analyses

After the eight-week experimental period, six eggs from each replicate were collected and divided into two groups of three. The first group was immediately evaluated for internal egg quality, and the second group was stored in a refrigerator (4 °C) for 30 days. Following the storage period, internal egg quality traits were assessed, including albumen height, Haugh unit, yolk height, yolk index, and the pH values of both albumen and yolk. Yolk diameter was measured using a digital caliper with 0.01 mm precision (BakingWin, China), and yolk index was calculated by dividing yolk height by Yolk diameter. Albumen height was determined with a manual Haugh Micrometer (Analog Baxlo Haugh Micrometer). For this, the egg was broken onto a flat surface, and albumen height and yolk height were measured by placing the gauge at specified points: 1 cm around the yolk for albumen height and directly on the yolk for yolk height. The following equation was used to calculate the Haugh unit (Haugh, 1937):

HU = 100 log (H + 7.57 - 1.7W^0.37^)

HU was Haugh unit, AH was egg albumen height (mm), EW was egg weight (g), and logarithm based on 10.

The Roche fan color scale was applied to evaluate the egg yolk color. To measure the pH of yolk and egg albumen, 2 g of egg albumen and egg yolk were mixed with distilled water at a ratio of 1:9 and stirred well until foam was formed (5 min). The pH value was recorded after subsiding the foam produced by inserting a pH meter sensor (Microcontroller MTT 65 model).

### 2.8 Fatty acid profile

In the case of yolk fatty acid profile, the yolk samples of 18 eggs per treatment (three eggs per replicate) were collected at week 8 of the experiment and stored at -20 °C for fatty acid profile determination. The yolk samples were extracted using the Soxhlet extraction method, homogenized by vortexing, and 100 mg of each sample was weighed. They were then saponified by adding 3 ml of 2 molar potassium hydroxide and esterified by adding 5 ml of 12 % methanolic sulfuric acid. The methyl ester of fatty acids was injected into the gas chromatography machine (Agilent 6890, Agilent CO, USA) using 0.1 ml of extracted normal heptane and 1 μl of normal heptane phase. The gas chromatography system was equipped with a capillary injector, a specialized capillary column for fatty acids (Stable Wax, 30 m length, 0.32 mm internal diameter, and 0.25 μm film thickness), and a flame ionization detector (FID). The initial oven temperature was set at 75 °C for one minute and gradually increased to 240 °C (25 °C per min) and maintained for 8 min (Yoon et al., 2024). Nitrogen gas (99.999 % purity, provided by Oxygen Sabalan Co. [the UK 108 Air Product Company’s agent]) was used as a carrier at a flow rate of 3 ml/min. The injector temperature was set at 250 °C and the detector at 280 °C. A mixture of all fatty acid**s**’ standards was also injected, and its retention time was used for comparison with egg yolk fatty acids (Mannion et al., 2016). The results of the retention time comparison were analyzed using Chemstation software in the Windows environment.

### 2.9 Statistical analysis

Data normality was tested with UNIVARIATE method and Shapiro-Wilk SAS 9.4 test. The data from this experiment were statistically analyzed in the form of a 2 × 3 factorial test using SAS software and the Mixed procedure (SAS, 2009).

y_ijk_ = µ + A_i_ + B_j_ + AB_ij_ + e_ijk_

In the above equation, µ was the total mean, A_i_ was the effect of the i-th level of flaxseed oil, B_j_ was the effect of the j-th level of SP, AB_ij_ was the interaction effect of the levels of flaxseed oil and SP, and e_ijk_ was the effect of experimental error or unknown factors in each observation. The significance of the differences between the data means was examined using Duncan's test at a probability level of 5 %.

A three-way ANOVA (flaxseed oil × SP × storage) was used to analyze the egg quality data.

y_ijk_ = µ + A_i_ + B_j_ +C_l_+ (AB)_ij_ + (AC)_il_+ (BC)_jl_+ (ABC)_ijl_ + e_ijle_

In the above equation, y_ijk_: value of the trait of interest, µ: total mean, A_i_: effect of the i-th flaxseed oil level, B_j_: effect of the j-th SP level, C_l_: effect of the l-th storage level, (AB)_ij_: interaction of flaxseed oil and SP levels, (AC)_il_: interaction of flaxseed oil and storage levels, (BC)_jl_: interaction of SP and storage levels, (ABC)_ijl_: interaction of flaxseed oil and SP and storage levels, and e_ijle_: was the effect of experimental error or unknown factors in each observation.

## 3 Results

### 3.1 Production performance

Table 2 shows the effect of using SP in diets containing different levels of flaxseed oil on the performance of laying hens. The interaction effects of SP and flaxseed oil showed that hens treated with 3 % flaxseed oil with or without SP had the highest percentage of production, weight, and mass of eggs, which were significantly higher than those treated without flaxseed oil (*P* < 0.05). The use of 3 % flaxseed oil with 5 g of SP reduced the FCR compared to other experimental treatments (*P* < 0.05). The results of feed cost per kilogram of egg production showed that the use of diets containing 1.5 and 3 % flaxseed oil with 5 g/kg of SP and diets containing 3 % flaxseed oil without SP increased feed cost per kilogram of egg despite improving egg mass (*P* < 0.05).

**Table 2:** Effects of using *Spirulina Platensis* in diets containing different levels of flaxseed oil on the performance of laying hens.

Treatment	EP (%)	EW (g)	EM (g/d/bird)	FI (g/d/bird)	FCR	Cost ($)
Flaxseed oil (%)						
0	80.58^b^	58.76^c^	47.41^c^	98.85	2.13^a^	0.693^b^
1.5	83.26^a^	59.76^b^	49.23^b^	99.10	2.03^b^	0.715^ab^
3	83.41^a^	61.26^a^	50.81^a^	98.95	1.96^c^	0.744^a^
SEM	0.68	0.29	0.37	1.13	0.02	0.009
*P-*Value	0.005	0.01	0.02	0.44	0.01	0.02
Spirulina (g/kg)						
0	81.64^b^	59.50^b^	48.33^b^	98.86	2.09^a^	0.704^b^
5	83.19^a^	60.36^a^	49.97^a^	99.06	1.99^b^	0.730^a^
SEM	0.39	0.23	0.30	1.11	0.02	0.009
*P-*Value	0.01	0.02	0.01	0.21	0.005	0.01
Flaxseed oil × Spirulina						
0 × 0	80.48^b^	58.67^b^	47.28^c^	98.90	2.22^a^	0.687^b^
0 × 5	80.69^b^	58.86^b^	47.55^b^	98.80	2.12^a^	0.698^b^
1.5 × 0	81.93^ab^	59.22^ab^	48.21^bc^	98.90	2.08^ab^	0.697^b^
1.5 × 5	84.58^ab^	60.29^a^	50.24^bc^	99.30	2.00^ab^	0.733^a^
3 × 0	82.52^a^	60.59^a^	49.50^b^	98.80	2.02^ab^	0.729^a^
3 × 5	84.31^a^	61.92^a^	52.12^a^	99.10	1.92^b^	0.760^a^
SEM	0.72	0.41	0.53	1.19	0.05	0.011
*P-*Value	0.02	0.003	0.008	0.40	0.01	0.001

*Note:* EP: Egg production; EW: Egg weight; EM: Egg mass; FI: Feed intake; FCR: Feed conversion ratio; Cost: Feed cost per kilogram of eggs produced; SEM: standard error of means.

^a-c^Means in the same column with different superscripts are significantly different (*P* < 0.05).

### 3.2 Antioxidant status

The effects of dietary SP supplementation at different flaxseed oil inclusion levels on liver and serum antioxidant parameters in laying hens are presented in Table 3. The interaction between SP and flaxseed oil levels revealed that diets containing 1.5 % and 3 % flaxseed oil significantly decreased TAC and liver SOD activity, as well as serum TAC (*P* < 0.05). However, the inclusion of 5 g/kg SP in these diets significantly increased these antioxidant indices (*P* < 0.05). Furthermore, serum MDA levels were significantly elevated in hens fed diets containing 1.5 % and 3 % flaxseed oil without SP supplementation, while dietary inclusion of SP mitigated this increase and reduced serum MDA concentrations even below those of the control group (*P* < 0.05).

**Table 3:** Effects of using *Spirulina Platensis* in diets containing different levels of flaxseed oil on antioxidant parameters of liver and blood serum.

	Blood		Liver			
Treatment	TAC (mmol/mL)	MDA (nmol/mL)	TAC (mmol/ g tissue)	MDA (nmol/ g tissue)	GSH-Px (U/mg protein)	SOD (U/mg protein)
Flaxseed oil (%)						
0	1.39	2.22	1.30	0.31^b^	4.95	15.61
1.5	1.30	2.37	1.06	0.35^ab^	5.18	16.01
3	1.29	2.50	1.17	0.38^a^	5.17	15.12
SEM	0.04	0.10	0.07	0.02	0.17	0.40
*P-*Value	0.62	0.52	0.11	0.03	0.30	0.27
Spirulina (g/kg)						
0	1.18^b^	2.78^a^	1.01^b^	0.37	4.77^b^	14.42^b^
5	1.31^a^	1.89^b^	1.31^a^	0.33	5.46^a^	17.32^a^
SEM	0.01	0.04	0.03	0.01	0.13	0.21
*P-*Value	0.01	0.02	0.01	0.07	0.02	0.01
Flaxseed oil × Spirulina						
0 × 0	1.33^ab^	2.36^ab^	1.18^ab^	0.33	4.50	14.29^b^
0 × 5	1.47^a^	1.94^b^	1.41^a^	0.29	5.30	17.98^a^
1.5 × 0	1.21^b^	2.72^a^	0.96^b^	0.34	4.87	13.02^b^
1.5 × 5	1.36^a^	1.97^b^	1.17^ab^	0.35	5.48	14.77^b^
3 × 0	1.16^b^	3.24^a^	0.98^b^	0.41	4.95	13.72^b^
3 × 5	1.38^a^	1.77^b^	1.37^a^	0.36	5.58	16.71^a^
SEM	0.06	0.09	0.05	0.02	0.24	0.33
*P-*Value	0.03	0.03	0.01	0.16	0.20	0.04

*Note:* TAC: total antioxidant capacity; MDA: malondialdehyde; GSH-Px: glutathione peroxidase; SOD: superoxide dismutase; SEM: standard error of means.

^a-c^Means in the same column with different superscripts are significantly different (*P* < 0.05).

### 3.3 Ovarian follicles

The main and interactive effects of different levels of SP and flaxseed oil supplementation on ovarian parameters are shown in Table 4. The interactive effects indicated that SP supplementation in 1.5 and 3 % flaxseed oil-containing diets had a synergistic effect on LYF weight, resulting in a significant increase compared to without SP diets (*P* < 0.05). Additionally, the number of LYF were significantly increased by the inclusion of 1.5 % and 3 % flaxseed oil in the diet compared to the control group (*P* < 0.01).

**Table 4:** Effects of using *Spirulina Platensis* in diets containing different levels of flaxseed oil on ovarian follicles in laying hens.

Treatment	Ovary (%BW)	Oviduct (%BW)	LYF number	SYF number	LYF (g)	SYF (g)	F1 (g)
Flaxseed oil (%)							
0	2.78	2.96	4.70^b^	9.30	30.10^b^	4.40	16.30
1.5	2.96	2.76	5.30^a^	8.90	34.00^a^	4.40	17.00
3	3.08	2.91	5.40^a^	8.40	36.60^a^	4.80	17.00
SEM	0.12	0.11	0.21	0.33	0.81	0.34	0.50
*P-*Value	0.15	0.51	0.01	0.11	0.02	0.55	0.46
Spirulina (g/kg)							
0	2.90	2.89	4.96	8.93	31.60^b^	4.50	16.39
5	2.97	2.85	5.30	8.80	35.13^a^	4.56	16.60
SEM	0.12	0.11	0.21	0.27	0.65	0.22	0.31
*P-*Value	0.64	0.82	0.31	0.89	0.03	0.87	0.35
Flaxseed oil × Spirulina							
0 × 0	2.74	3.03	4.50	9.40	28.20^c^	4.70	16.40
0 × 5	2.81	2.88	4.90	9.00	32.00^b^	4.10	16.20
1.5 × 0	2.87	2.72	5.10	9.20	31.60^b^	4.10	17.40
1.5 × 5	3.04	2.81	5.50	8.60	36.40^a^	4.70	16.60
3 × 0	3.10	2.94	5.30	8.20	35.00^a^	4.70	17.00
3 × 5	3.06	2.88	5.50	8.80	37.00^a^	4.90	17.00
SEM	0.28	0.29	0.48	0.52	1.35	0.32	0.51
*P-*Value	0.23	0.37	0.93	0.57	0.01	0.28	0.81

*Note:* BW: body weight; LYF: large yellow follicle; SYF: small yellow follicle; F1: the largest follicle ready for ovulation; SEM: standard error of means.

^a-c^Means in the same column with different superscripts are significantly different (*P* < 0.05).

### 3.4 Egg storage

The main and interactive effects of SP and flaxseed oil supplementation on egg shelf life during storage are summarized in Table 5. The interaction effects of SP and flaxseed oil showed that the use of 5 g/kg SP in diets containing 1.5 and 3 % flaxseed oil significantly reduced the Haugh unit compared to diets containing 1.5 and 3 % flaxseed oil without SP (*P* < 0.01). The main effects demonstrated that increasing levels of dietary flaxseed oil significantly reduced yolk index in fresh eggs. However, SP supplementation significantly improved yolk color and Haugh unit, and also reduced albumen pH (*P* < 0.05). Egg storage for 30 days led to a decrease in Haugh unit, yolk index, and yolk color, accompanied by an increase in albumen and yolk pH. The three-way interaction (flaxseed oil × SP × storage) revealed that SP supplementation significantly improved Haugh unit values during storage compared to treatments without SP (*P* < 0.05).

**Table 5:** Effects of using *Spirulina Platensis* in diets containing different levels of flaxseed oil on egg quality during storage.

Treatment	Haugh unit	Yolk index	Yolk pH	Albumen pH	Yolk color
Flaxseed oil (%)					
0	81.05^a^	32.35^a^	6.44	8.75	5.77
1.5	80.15^b^	30.65^b^	6.26	8.59	7.91
3	79.60^b^	31.20^b^	6.40	8.66	7.89
SEM	0.38	0.28	0.10	0.12	0.09
P-Value	0.02	0.03	0.29	0.48	0.31
Spirulina (g/kg)					
0	80.68^b^	32.00	6.37	9.02^a^	7.48^b^
5	81.99^a^	32.13	6.36	8.40^b^	8.23^a^
SEM	0.31	0.23	0.08	0.11	0.09
*P-*Value	0.03	0.43	0.75	0.01	0.03
Storage time (days)					
0	89.45^a^	32.66^a^	6.12^b^	7.97^b^	7.98^a^
30	73.22^b^	31.46^b^	6.59^a^	9.45^a^	7.53^b^
SEM	0.31	0.23	0.08	0.11	0.09
*P-*Value	0.003	0.001	0.027	0.016	0.008
Flaxseed oil × Spirulina × Storage					
0 × 0 × 0	79.30^b^	32.80	6.23	8.21	7.69
0 × 0 × 30	70.39^d^	29.60	6.85	9.99	7.13
0 × 5 × 0	89.63^a^	32.00	6.13	7.99	8.29
0 × 5 × 30	72.90^c^	31.00	6.54	8.82	7.97
1.5 × 0 × 0	90.77^a^	33.20	5.96	7.74	7.77
1.5 × 0 × 30	73.60^c^	32.00	6.32	10.18	7.24
1.5 × 5 × 0	91.00^a^	33.20	6.07	8.08	8.52
1.5 × 5 × 30	74.05^c^	32.20	6.68	8.99	8.11
3 × 0 × 0	88.53^a^	33.00	6.15	7.99	7.74
3 × 0 × 30	73.53^c^	31.40	6.70	10.08	7.31
3 × 5 × 0	89.49^a^	31.80	6.26	7.91	8.47
3 × 5 × 30	74.85^c^	32.60	6.48	8.65	7.85
SEM	0.77	0.57	0.39	0.34	0.13
*P-*Value	0.01	0.24	0.75	0.43	0.19

*Note:* SEM: standard error of means.

^a-c^Means in the same column with different superscripts are significantly different (*P* < 0.05).

### 3.5 Yolk fatty acid profile

The main and interactive effects of different levels of SP supplementation and flaxseed oil on the saturated fatty acids (**SFAs**) and monounsaturated fatty acids (**MUFAs**) profile of egg yolk—specifically myristic acid, myristoleic acid, palmitic acid, palmitoleic acid, stearic acid, and oleic acid—are presented in Table 6. The results showed that the interaction between 3 % flaxseed oil and 5 g/kg SP supplementation significantly enhanced the proportion of oleic acid in egg yolk (*P* = 0.034). The dietary inclusion of 1.5 % and 3 % flaxseed oil significantly increased the proportions of myristic and palmitoleic acids while reducing palmitic and stearic acid levels (*P* < 0.05). Furthermore, supplementation with SP at 5 g/kg significantly decreased palmitoleic acid concentrations (*P* = 0.008).

**Table 6:** Effects of using *Spirulina Pl*a*tensis* supplement in diets containing different levels of flaxseed oil on the percentage of saturated and unsaturated fatty acids with one double bond in egg yolk.

Treatment	Myristic (C14:0)	Myristoleic (C14:1)	Palmitic (C16:0)	Palmitoleic (C16:1)	Stearic (C180:0)	Oleic (C18:1)
Flaxseed oil (%)						
0	0.339^b^	0.162	24.92^a^	2.23^c^	12.10^a^	47.62^b^
1.5	0.395^a^	0.111	24.86^a^	3.02^b^	10.22^b^	47.69^b^
3	0.398^a^	0.063	24.03^b^	3.42^a^	9.11^c^	48.82^a^
SEM	0.005	0.034	0.222	0.042	0.081	0.176
*P-*Value	0.005	0.531	0.041	0.028	0.009	0.002
Spirulina (g/kg)						
0	0.384	0.144	25.12^a^	2.84	10.43	47.68^b^
5	0.372	0.065	24.18^b^	2.91	10.52	48.35^a^
SEM	0.005	0.034	0.222	0.042	0.081	0.176
*P-*Value	0.361	0.508	0.008	0.470	0.431	0.047
Flaxseed oil × Spirulina						
0 × 0	0.384	0.228	24.93	2.19	12.03	47.66^b^
0 × 5	0.332	0.085	24.69	2.26	12.19	47.64^b^
1.5 × 0	0.404	0.146	25.78	2.99	10.16	46.84^b^
1.5 × 5	0.390	0.058	24.26	3.01	10.32	48.34^ab^
3 × 0	0.402	0.058	24.56	3.35	9.10	48.55^ab^
3 × 5	0.396	0.052	23.59	3.47	9.12	49.05^a^
SEM	0.009	0.061	0.385	0.073	0.140	0.305
*P-*Value	0.942	0.681	0.517	0.734	0.518	0.034

*Note:* SEM: standard error of means.

^a-c^Means in the same column with different superscripts are significantly different (*P* < 0.05).

The effects of SP and flaxseed oil on PUFAs, including omega-3 and omega-6 fatty acids, are shown in Table 7. The interaction effects of SP and flaxseed oil showed that the use of 5 g/kg of SP in diets containing 3 % flaxseed oil significantly increased the content of α-linolenic acid, EPA, and DHA compared to other treatments (*P* < 0.01). The increasing flaxseed oil levels (to 1.5 % and 3 %) significantly reduced linoleic acid content in the egg yolk compared to the control (*P* < 0.05).

**Table 7:** Effects of using *Spirulina Platensis* supplement in diets containing different levels of flaxseed oil on the polyunsaturated fatty acids profile of egg yolk at the end of the period.

Treatment	Linoleic acid (C18:2 n-6)	Arachidonic acid (C20:4 n-6)	α-linolenic acid (C18:3 n-3)	EPA (C20:5 n-3)	DHA (C22:6 n-3)
Flaxseed oil (%)					
0	10.85^a^	0.157^c^	0.898^c^	0.148^c^	0.288^c^
1.5	10.03^b^	0.617^b^	0.988^b^	0.581^b^	1.219^b^
3	9.56^c^	0.813^a^	1.061^a^	1.006^a^	1.540^a^
SEM	0.081	0.011	0.011	0.014	0.006
*P-*Value	0.037	0.001	0.006	0.007	0.026
Spirulina (g/kg)					
0	10.06	0.528	0.955^b^	0.538	0.981^b^
5	10.21	0.536	1.027^a^	0.605	1.042^a^
SEM	0.081	0.011	0.011	0.014	0.006
*P-*Value	0.520	0.815	0.002	0.067	0.049
Flaxseed oil × Spirulina					
0 × 0	10.79	0.156	0.878^c^	0.140^d^	0.282^d^
0 × 5	10.98	0.164	0.914^c^	0.152^d^	0.304^d^
1.5 × 0	9.97	0.624	0.984^b^	0.560^c^	1.188^c^
1.5 × 5	10.03	0.620	0.994^b^	0.592^c^	1.224^c^
3 × 0	9.42	0.806	1.004^a^	0.914^b^	1.470^b^
3 × 5	9.60	0.824	1.174^a^	1.072^a^	1.598^a^
SEM	0.141	0.019	0.018	0.024	0.010
*P*-Value	0.716	0.248	0.003	0.001	0.003

*Note:* EPA: Eicosapentaenoic acid; DHA: Docosaheaenoic acid; SEM: standard error of means.

^a-c^Means in the same column with different superscripts are significantly different (*P* < 0.05).

The main and interactive effects of flaxseed oil and SP on fatty acid ratios in egg yolk are shown in Table 8. A significant interaction was observed, showing that combined inclusion of flaxseed oil and SP led to a further increase in yolk omega-3 fatty acid content (*P* = 0.027). Moreover, the linoleic acid to α-linolenic acid ratio was significantly reduced by the combined effect of flaxseed oil and SP supplementation in the diet (*P* = 0.002). The inclusion of flaxseed oil significantly increased total PUFA content, the PUFA/SFA ratio, and the omega-3 fatty acid content in a dose-dependent manner (*P* < 0.05), while total SFA content decreased linearly. Conversely, omega-6 fatty acid levels and the omega-6/omega-3 ratio significantly declined with increasing flaxseed oil levels, as expected (*P* < 0.05).

**Table 8:** Effects of *Spirulina Platensis* supplementation in diets containing different levels of flaxseed oil on fatty acid ratios in egg yolk.

Treatment	SFA	PUFA	PUFA/ SFA	∑ n-3	∑ n-6	n-6: n-3	LA/ALA
Flaxseed oil (%)							
0	37.37^a^	12.34^c^	0.331^b^	1.33^c^	11.09^a^	8.25^a^	12.08^a^
1.5	35.49^b^	13.42^b^	0.378^ab^	2.77^b^	10.64^b^	3.93^b^	10.15^b^
3	33.54^c^	13.97^a^	0.416^a^	3.60^a^	10.37^c^	2.88^c^	9.08^c^
SEM	0.380	0.088	0.004	0.016	0.083	0.038	0.127
P-Value	0.016	0.001	0.003	0.034	0.008	0.026	0.003
Spirulina (g/kg)							
0	35.94	13.06^b^	0.364^b^	2.47^b^	10.59	5.11^a^	10.62^a^
5	35.10	13.41^a^	0.384^a^	2.67^a^	10.74	4.88^b^	10.10^b^
SEM	0.381	0.088	0.004	0.016	0.083	0.038	0.127
P-Value	0.281	0.009	0.016	0.042	0.571	0.037	0.001
Flaxseed oil × Spirulina							
0 × 0	37.31	12.25	0.328	1.30^c^	10.95	8.43	12.30^a^
0 × 5	37.22	12.51	0.336	1.37^c^	11.14	8.13	12.01^a^
1.5 × 0	36.35	13.33	0.368	2.73^b^	10.59	3.88	10.16^b^
1.5 × 5	34.97	13.46	0.386	2.81^b^	10.65	3.79	10.10^b^
3 × 0	24.15	13.61	0.396	3.38^a^	10.22	3.02	9.40^c^
3 × 5	33.10	14.26	0.432	3.84^a^	10.42	2.71	8.18^d^
SEM	0.312	0.153	0.006	0.028	0.145	0.066	0.219
*P-*Value	0.234	0.384	0.308	0.027	0.719	0.346	0.002

*Note:* PUFA: polyunsaturated fatty acids; SFA: saturated fatty acids; LA: linoleic acid; ALA: α-linolenic acid; SEM: standard error of means.

^a-c^Means in the same column with different superscripts are significantly different (*P* < 0.05).

### 3.6 Relationship between MDA and DHA and meeting daily omega-3 requirement

**Fig. 1:**
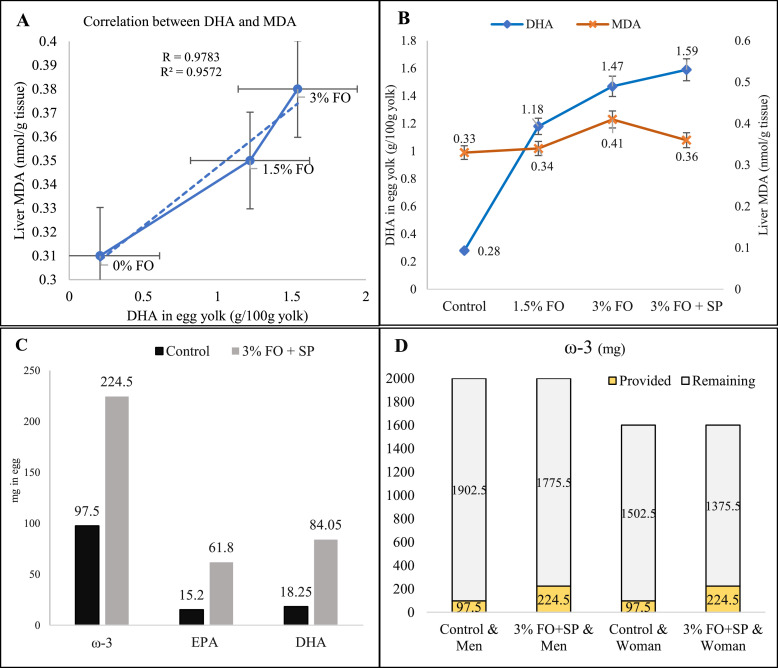
Relationship between egg yolk docosahexaenoic acid (DHA) and hepatic malondialdehyde (MDA) levels, as well as the contribution of conventional and enriched eggs to meeting the daily omega-3 requirements. (**A**) Correlation between yolk DHA concentration and liver tissue MDA levels in laying hens fed flaxseed oil (FO) diets (0 %, 1.5 %, and 3 %). (**B**) Effect of Spirulina (SP) on the relationship between yolk DHA content and liver MDA levels in hens fed 3 % FO. (**C**) Comparison of omega-3, eicosapentaenoic acid (EPA), and DHA contents (mg/egg) in control eggs and eggs enriched with 3 % FO plus SP. (**D**) Contribution of control and enriched eggs to meeting daily omega-3 requirements for adult men and women.

Fig. 1 illustrates the relationship between egg yolk DHA and hepatic MDA levels, as well as the contribution of conventional and enriched eggs to meeting the daily omega-3 requirements. The findings illustrated in Figure A demonstrate a clear positive correlation between DHA concentration in egg yolk and hepatic MDA levels in laying hens fed diets containing flaxseed oil. The correlation diagram showed that with an increase in DHA from 0.208 to 1.540 g per 100 g of yolk, the level of liver MDA increased linearly from 0.31 to 0.38 nmol/g of tissue. A linear regression line was fitted to the treatment means, revealing a strong positive Pearson correlation (r = 0.9783) and a high coefficient of determination (r² = 0.9572), indicating that DHA enrichment is strongly associated with increased liver lipid peroxidation in the absence of adequate antioxidant protection. In contrast, Figure B revealed that dietary supplementation with SP in combination with 3 % flaxseed oil resulted in a significant reduction of hepatic MDA levels, despite the concomitant increase in yolk DHA concentration. This highlights the antioxidant potential of SP in preventing lipid peroxidation and maintaining oxidative stability. Moreover, as shown in Figure C, hens fed the diet supplemented with 3 % flaxseed oil plus SP produced eggs with significantly higher contents of total omega-3 fatty acids, EPA, and DHA compared with the control group. Finally, Figure D illustrates the contribution of these enriched eggs to meeting the daily omega-3 requirements of adults. While a conventional egg supplied only one-twentieth and one-sixteenth of the daily omega-3 requirement for men and women, respectively, a single enriched egg provided approximately one-eighth of the daily requirement for men and one-seventh for women.

### 3.7 Omega-3 cost analysis

**Fig. 2:**
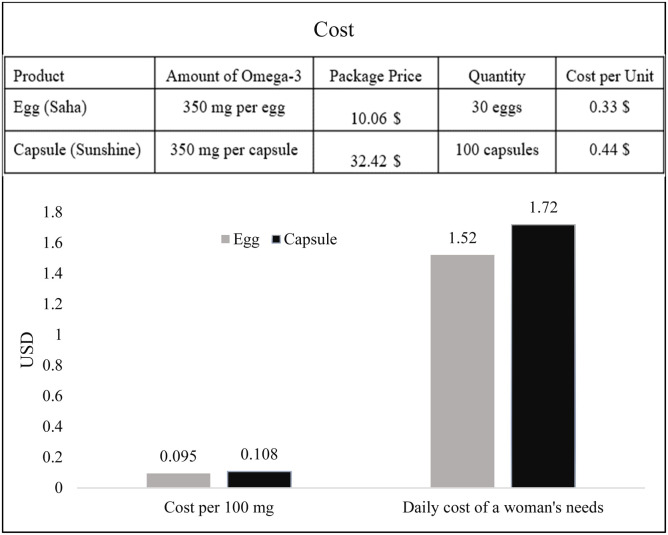
Comparison of omega-3 enriched eggs and commercial omega-3 capsules in terms of package price, cost per 100 mg of omega-3, and the daily cost of meeting a woman’s omega-3 requirement.

As shown in Fig. 2, both omega-3 enriched eggs and commercial fish oil capsules provided 350 mg of omega-3 per unit. However, the cost analysis revealed that enriched eggs were more economical. The cost per 100 mg of omega-3 was 0.095 USD for enriched eggs compared with 0.108 USD for capsules. More importantly, the daily cost of meeting a woman’s omega-3 requirement was estimated at 1.52 USD when provided through enriched eggs, whereas the equivalent intake through capsules required 1.72 USD. These findings indicate that omega-3 enriched eggs are a more cost-effective dietary source of omega-3 compared to conventional capsule supplementation, while also offering the additional benefits of being a natural and commonly consumed food.

## 4 Discussion

### 4.1 Production performance

In the present study, the addition of flaxseed oil to the diet of laying hens resulted in a significant improvement in production performance, as observed in increased egg production rate, egg weight, and improved FCR. In agreement with our results, researchers reported that flaxseed oil increased egg production and weight in laying hens (Attia et al., 2022). Similarly, Saleh et al. (2019) showed that daily egg production and egg mass in aged laying hens were significantly increased by supplementation with 1 g/kg flaxseed. In contrast, several studies reported that the addition of 2 to 3 % flaxseed oil to the diet of laying hens did not cause any significant change in production performance (Ceylan et al., 2011; Neijat et al., 2016). However, due to the presence of anti-nutritional factors in flaxseed oil, laying hens fed diets containing 5 % or higher levels of flaxseed oil had significantly reduced egg production (Kim et al., 2016). The discrepancy between the reported results may be due to variations in experimental diet composition, flaxseed oil level, age of birds, and feeding duration. flaxseed oil is a rich source of α-linolenic acid, which plays an important role in improving lipid metabolism, increasing energy efficiency, and modulating hormonal profiles related to ovulation and follicular development (Khatibjoo et al., 2018). The authors of the present study believe that the improvement in energy utilization efficiency and metabolism in birds fed flaxseed oil-containing diets likely resulted in better allocation of nutrients towards egg synthesis, ultimately leading to increased egg laying rate and egg weight. Also, the high levels of UFAs in flaxseed oil may improve the fluidity and function of cell membranes, especially in reproductive tissues, which could lead to better follicle response and increased reproductive system efficiency (Ayerza & Coates, 2001). It has been reported that the improved performance with diets containing vegetable oils could be due to the effect of these fat sources on the development of intestinal tissue and increased persistence of consumed food in the intestinal environment, as these conditions increase the digestion and absorption of nutrients (Shahryari et al., 2021). On the other hand, the reason for the increased performance could be related to the effect of omega-3 fatty acids in F, which improved follicle growth and weight, leading to improved production performance and increased egg weight. In addition, the protective role of omega-3 in preventing premature ovarian aging, especially in older laying hens, could also be effective in this improvement (Al-Safi et al., 2016).

As expected, SP supplementation had synergistic effects with flaxseed oil due to its antioxidant support, increasing egg production percentage, weight and mass. In agreement with the present results, researchers reported improvements in FCR, egg weight and egg production in hens fed a diet containing 2 % SP (Panaite et al., 2023). These positive effects of SP can be attributed to the high content of protein, essential amino acids, vitamins and minerals present in SP, as well as the presence of some bioactive compounds (bioactive compounds such as β-carotene and α-linolenic acid) and antioxidants (Anvar & Nowruzi, 2021). In a similar study (Salahuddin et al., 2024), laying hens fed 2.5 and 5 g/kg of SP improved egg weight and egg production, and the researchers reported that the nutrients in SP may stimulate the ovaries and promote the development of larger eggs, which is particularly beneficial for egg producers seeking to meet specific market size requirements.

### 4.2 Antioxidant status

The results of this study showed that the use of flaxseed oil in the diet of laying hens led to a significant increase in the concentration of MDA in the liver tissue. MDA is known as a reliable indicator of lipid peroxidation and oxidative stress, and its increase indicates increased oxidative damage of lipids in tissues. The increased content of MDA in the liver of birds fed a diet containing 3 % flaxseed oil is most likely related to the presence of higher concentrations of long-chain UFAs that are highly peroxidized. This finding indicates that although flaxseed oil is a rich source of omega-3 fatty acids (such as α-linolenic acid) and has high nutritional value, its consumption can increase the sensitivity of body tissues to oxidation (Goyens et al., 2006). Unsaturated fats, especially omega-3 PUFAs, are more susceptible to oxidation due to their multiple double bonds, and in the absence of adequate amounts of protective antioxidants, their degradation products such as MDA accumulate in tissues. The insignificant effect of flaxseed oil on serum MDA levels may indicate that serum MDA is maintained at normal levels by some protective mechanisms such as liver microsomes that are capable of producing and degrading MDA (Venkatraman et al., 1998). These findings may indicate that liver MDA levels are a more sensitive indicator of peroxidative propensity due to the abundance of substrates for thiobarbituric acid production in the liver and/or the accumulation of thiobarbituric acid produced in other transporters through the circulating blood. In the most recent studies, an increase in egg yolk MDA was observed due to an increase in UFAs, because UFAs are highly susceptible to oxidation, and therefore the amount of thiobarbituric acid compounds, or MDA, increased in the yolk of fresh eggs and eggs stored at 4 degrees Celsius for 28 days (Kralik et al., 2021).

The negative impact of lipid peroxidation in poultry is primarily associated with disturbances in redox homeostasis (Akbarian et al., 2016). The present findings demonstrated that the elevation in liver MDA concentration—a well-recognized marker of oxidative stress—was attenuated by the inclusion of SP in flaxseed oil-based diets. Simultaneously, the activities of GSH-Px and TAC were significantly improved in SP-supplemented groups. The ability of SP to restore redox balance in diets rich in PUFAs such as flaxseed oil can be attributed to its wide array of bioactive antioxidant compounds, including flavonoids, polyphenols, and other potent radical scavengers such as α-tocopherol, ascorbic acid, β-carotene, and selenium (Agustini et al., 2015). This indicates the potential for SP-derived antioxidant molecules to be absorbed and transferred into the bird's systemic circulation and liver tissue. Consistent with our results, Abdel-Moneim et al. (2022) and Panaite et al. (2023) reported that SP supplementation led to a significant improvement in the activities of key antioxidant enzymes, including SOD, catalase, and GSH-Px, as well as TAC. The observed reduction in liver MDA levels and enhancement in antioxidant enzyme activities in flaxseed oil-fed birds supplemented with SP further supports the hypothesis that these bioactive compounds can effectively protect hepatocytes against reactive oxygen species generated through lipid peroxidation (Akila et al., 1998). Therefore, the current study provides compelling evidence that SP supplementation can penetrate systemic circulation and liver tissues of laying hens and effectively counteract lipid peroxidation, thereby enhancing antioxidant capacity and preserving cellular integrity.

### 4.3 Ovarian follicles

The findings of the present study demonstrated that, irrespective of antioxidant supplementation, dietary flaxseed oil enhanced follicular growth, increased the number of follicles, elevated ovulation rates, and contributed to a higher ovarian weight. Nateghi et al. (2019) reported an increase in the number of LYF as well as the total number of follicles in laying hens fed flaxseed oil. Eilati et al. (2013) found that a three-week flaxseed oil supplementation improved egg production and downregulated the expression of Cyclooxygenase-1 and -2 mRNA compared to control groups. As the rate-limiting enzymes in the conversion of arachidonic acid to prostaglandins, which play critical roles in inflammatory and reproductive processes, Cyclooxygenase suppression by flaxseed oil suggests a potential mechanism for its reproductive benefits (Eilati et al., 2013). The results obtained in this study further support the hypothesis that omega-3 fatty acids, particularly DHA, are pivotal in regulating folliculogenesis. Importantly, this effect appeared to be independent of SP supplementation. Various mechanisms have been proposed to explain the impact of omega-3 fatty acids on ovarian function. It has been suggested that omega-3 fatty acids stimulate follicle-stimulating hormone (**FSH**), promoting follicular growth, maturation, and ovulation (Bender et al., 2010). Among the key bioactive components, EPA and DHA have been shown to enhance ovulation and improve ovarian blood flow, contributing to follicle growth and increased ovarian weight (Senger, 2005). Moreover, omega-3 fatty acids are believed to exert their reproductive effects primarily through hypothalamic receptors, modulating the release of gonadotropin-releasing hormone, which subsequently stimulates FSH and luteinizing hormone (**LH**) secretion (Watanabe, 2002). On the other hand, PUFAs in flaxseed oil increase the production of estradiol and progesterone and facilitate the process of folliculogenesis by modulating the expression of genes related to steroidogenesis. In the present study, the weight of LYF was significantly higher in the flaxseed oil-containing treatments, which is probably due to the increase in lipid reserves in the follicle and the improvement in the metabolic status of follicular cells. Lipids, as the main source of energy and structural components of cell membranes, play a key role in follicle growth and steroid hormone production (Cherian, 2008).

In addition, SP, due to its high-quality protein, essential amino acids and minerals such as iron and zinc, can improve follicular development and ovarian function by improving the nutritional status of the bird. Some studies have shown that SP consumption can increase estradiol and progesterone levels, thereby positively affecting development and ovulation (Abouelezz, 2017). Similar studies have also shown that the addition of flaxseed oil and SP to the diet of laying hens improved egg quality traits, increased production, and reduced the rate of yolkless eggs (Attia et al., 2022; Hajati et al., 2024), all of which confirm the positive effects of these compounds on reproductive performance. From a physiological perspective, any increase in the number and weight of large yolk follicles can mean improved oviposition performance and increased egg-laying capacity in laying hens. Also, larger follicles usually contain higher amounts of lipid and protein, which can also have a positive effect on egg weight, egg quality and the health of the resulting chicks.

### 4.4 Egg storage

Long-term storage of eggs is one of the factors affecting the decline in internal quality and physical and chemical properties of eggs. In the present study, eggs were stored at refrigerator temperature for 30 days and then their quality traits were evaluated. The results showed that the storage period caused significant and undesirable changes in the internal parts of eggs. The decline in the quality of albumen and yolk due to long-term storage at room temperature is a well-known phenomenon that occurs mainly due to the evaporation of carbon dioxide from the egg, followed by changes in internal pH, protein decomposition and denaturation, and water transfer between different parts of the egg (Jin et al., 2010). After 30 days of storage, a significant decrease in the value of Howe units was observed, indicating a decrease in the consistency of egg white. This quality decline is mainly due to the increase in the pH of the albumen and, as a result, the denaturation and loss of the natural structure of the main albumen proteins such as ovalbumin, ovotransferrin and lysozyme (Doğan et al., 2018). With an increase in pH above 9 during storage, the hydrogen bonds between the protein chains of the albumen are weakened and its gel structure is broken. On the other hand, the release of CO₂ from the air chamber of the egg is the main factor in the increase in pH and the change in the consistency of the albumen during storage. These changes lead to a decrease in the viscosity and gelling properties of the albumen and ultimately cause a decrease in the quality value of the egg (Li et al., 2015). In this regard, pH changes in the internal parts of the egg were also significant; so that the pH of the albumen increased significantly during storage, while the pH of the yolk showed a slight decrease. In addition to denaturing proteins, increasing the pH of the egg white increases the permeability of the yolk membrane, which results in more water being transferred from the egg white to the yolk. This transfer of water into the yolk causes it to swell and dilute, and destroys the lipid-protein structure of the yolk. Such changes not only cause a decrease in yolk consistency, but also negatively affect the yolk index (the ratio of yolk height to diameter) (Huang et al., 2022). The decrease in yolk index due to storage of eggs at ambient temperature is a reported phenomenon that occurs due to an increase in the water volume of the yolk, the breakdown of the lipoprotein structure, and the oxidation of its fats during storage. In fact, an increase in the permeability of the yolk membrane and the leakage of water and salts into it are among the factors affecting the reduction of yolk quality (Bosch et al., 2021).

Another notable finding in this study was the ability of SP to reduce the negative effects of long-term egg storage. The results showed that the use of SP in the diet of laying hens resulted in better preservation of internal egg quality during the storage period. In particular, eggs produced by hens that received SP experienced a lower decrease in Howe's unit and yolk index compared to the control group. The reason for this protective function can be attributed to the antioxidant and anti-inflammatory compounds of SP. SP contains carotenoids, phycocyanin and phenolics that are able to protect against the oxidation of yolk lipids and the denaturation of albumen proteins against high pH and oxidation. In addition, by preserving the yolk membrane and reducing its permeability to water and salts, SP limits the migration of liquid from the albumen to the yolk, and consequently reduces the decline in the yolk index during storage (Salahuddin et al., 2024). Overall, these findings indicate that storing eggs at refrigerated temperature for 30 days significantly reduces the internal quality of eggs through an increase in albumen pH, a decrease in yolk pH, a decrease in albumen consistency, a decrease in Howe's units, and a decrease in the yolk index. However, adding SP to the diet of laying hens can significantly compensate for the negative effects of storage and maintain egg quality during storage.

### 4.5 Yolk fatty acid profile

In this study, the evaluation of yolk fatty acid profiles in laying hens fed diets supplemented with flaxseed oil revealed an increase in PUFAs, particularly omega-3 fatty acids, including EPA, DHA, and α-linolenic acid. Conversely, levels of linoleic acid, the omega-6 to omega-3 ratio, and the linoleic acid to α-linolenic acid ratio were significantly reduced compared to the control group. These findings are consistent with previous studies that reported alterations in the fatty acid profile of eggs from hens fed flaxseed oil-supplemented diets. In the meta-analysis by Irawan et al. (2022), increasing the level of dietary α-linolenic acid led to elevated concentrations of EPA, DHA, and total omega-3 PUFAs in egg yolk, while simultaneously decreasing linoleic acid levels. In a similar study, a 30.5 % increase in α-linolenic acid levels in the yolk was observed in laying hens fed diets containing 3.5 % fish oil, compared to birds fed a control diet (Saleh, 2013). These results suggest that feeding hens a diet containing 3 % flaxseed oil may result in eggs enriched with a higher proportion of DHA. The total omega-3 PUFA content of the diet can also serve as a predictor of DHA formation, since α-linolenic acid is the principal precursor of omega-3 fatty acids in most dietary fat sources. Our findings align with the established theory that α-linolenic acid acts as the main precursor for the synthesis of EPA and DHA. In laying hens, dietary α-linolenic acid is desaturated by Δ-6-desaturase through the removal of hydrogen atoms and is subsequently elongated by the addition of carbon atoms to form EPA. This EPA is then further elongated and desaturated to form DHA (Fraeye et al., 2012). The efficiency of α-linolenic acid conversion to DHA varies across experimental conditions, primarily due to differences in the dietary linoleic acid content (Ehr et al., 2017). This is because Δ-6-desaturase can also act on linoleic acid as a substrate, albeit with lower activity compared to α-linolenic acid. Therefore, an increased linoleic acid / α-linolenic acid ratio may hinder DHA synthesis in eggs due to substrate competition (Fraeye et al., 2012). Several recent studies support this mechanism, reporting that lowering the dietary linoleic acid / α-linolenic acid ratio from various fat sources linearly increases DHA deposition in egg yolk (Aguillón-Páez et al., 2020; Lee et al., 2021). In addition to the linoleic acid/α-linolenic acid ratio, other factors influencing DHA deposition include the age of the hens and the source of dietary fatty acids. Older hens, due to having larger livers, may exhibit more efficient metabolism—especially in converting α-linolenic acid to DHA (Fraeye et al., 2012).

Most importantly, it should be considered that one of the major challenges in incorporating omega-3 fatty acids into diets is their high susceptibility to lipid oxidation (Faitarone et al., 2016), which directly affects the quality and flavor of eggs. A previous study using fish oil and flaxseed oil reported an increase in lipid oxidation in eggs (Kralik et al., 2021). Therefore, the addition of antioxidants alongside omega-3 fatty acid sources has been proposed as an effective strategy to inhibit oxidation, potentially extending egg shelf life (Huang et al., 2019; Omri et al., 2019). There is limited information on the potential effects of SP supplementation on yolk fatty acid composition. Some authors, using very high doses (5 %) and longer supplementation periods, observed an increase in MUFA and a reduction in PUFA (Boiago et al., 2019), whereas other researchers reported increased levels of linoleic and α-linolenic acid with similar dosages (Rajesha et al., 2009). In other studies, supplementation for more than 30 days led to an increase in PUFA levels. More data are needed to clarify fatty acid changes. The findings of Rey et al. (2010) suggest that longer supplementation periods or higher doses may be necessary to enhance the content of key fatty acids such as omega-3. In the present study, a very strong positive linear correlation (r = 0.978, r² = 0.957) was observed between yolk DHA levels and liver MDA concentrations at different levels of flaxseed oil (0, 1.5 and 3 %) (Fig. 1). This finding suggests that although flaxseed oil supplementation effectively increases omega-3 fatty acids, especially DHA, in the yolk, it may also lead to increased lipid peroxidation in liver tissue. These results demonstrate the high vulnerability of long-chain unsaturated fatty acids to oxidative damage and justify the use of natural antioxidant sources such *As sp* in PUFA-enriched diets. However, there is limited data regarding the effects of SP on egg fat stability. Some studies have demonstrated an increase in total antioxidant potential in the serum of hens fed diets supplemented with 2 % SP for 12 weeks (Tufarelli et al., 2021). Additionally, Mirzaie et al. (2018) reported the efficacy of SP in enhancing antioxidant status by increasing antioxidant enzymes or reducing MDA in broilers exposed to heat stress. However, measurements in these studies were taken from serum/plasma of live animals rather than tissue samples. Studies examining the potential effects of SP supplementation on animal tissues are scarce. Miranda et al. (1998) observed lower MDA levels in brain homogenates of mice supplemented with SP for 2 or 7 weeks compared to controls. In a unique study on egg fat stability, Boiago et al. (2019) reported lower lipid peroxidation levels in yolks of quails fed at least 5 % SP for 42 days. Therefore, more information is needed to determine the effects of this nutrient on yolk fatty acid profiles and stability. The results of the present study suggest that SP supplementation for 8 weeks may serve as a promising strategy to improve the fatty acid profile of egg yolk.

Although the present study provides valuable insights into the synergistic effects of SP and flaxseed oil on production performance, antioxidant capacity, ovarian development, and egg shelf life in laying hens, certain aspects warrant further investigation. The current experiment mainly focused on physiological and biochemical parameters; therefore, exploring molecular mechanisms underlying the antioxidant and reproductive responses to SP supplementation and flaxseed oil would strengthen future studies.

## 5 Conclusions

The findings of this study showed that adding flaxseed oil to the diet of laying hens, while improving production performance, increased lipid peroxidation and reduced egg stability during storage. However, simultaneous supplementation with SP not only improved production performance and ovarian indices, but also increased antioxidant status and egg shelf life. The combination of these two sources produced eggs with an improved lipid profile that was rich in beneficial omega-3 fatty acids and reduced in harmful fatty acids. Accordingly, the combined use of SP and flaxseed oil can be recommended as a practical strategy for producing functional and healthier eggs.

## Ethical statement

### Dear editor-in-chief of the veterinary and animal science

With all respects and thanks for your kindness. The authors of this manuscript are obliged to remined the following points:

1. The authors confirm that the ethical policies of the journal, as noted on the journal’s author guidelines page, have been adhered to and the appropriate ethical review committee approval has been received (In line with the Animal Research Ethical Committee of the Animal Science Department of Urmia University under the ethical approval number of IR-UU-AEC-3/116). The authors confirm that they have followed EU standards for the protection of animals used for scientific purposes.

## CRediT authorship contribution statement

**Javad Ghanouni-Bostanabad:** Writing – original draft, Visualization, Investigation. **Seyyed Ali Mirghelenj:** Writing – review & editing, Visualization, Validation, Project administration. **Mohsen Daneshyar:** Writing – review & editing, Resources, Data curation. **Ali Hashemi:** Writing – review & editing, Project administration, Methodology. **Sina Payvastegan:** Writing – review & editing, Supervision, Investigation, Funding acquisition. **Amir Mosayyeb Zadeh:** Visualization, Validation, Project administration. **Hamzeh Ghaderi-Chaparabad:** Visualization, Validation, Software, Conceptualization. **Motaleb Ebrahimi:** Writing – review & editing, Software, Formal analysis, Data curation.

## Declaration of competing interest

The authors declare that they have no known competing financial interests or personal relationships that could have appeared to influence the work reported in this paper.

## References

[bib0001] Abdel-Moneim A.M.E., Shehata A.M., Selim D.A., El-Saadony M.T., Mesalam N.M., Saleh A.A. (2022). Spirulina platensis and biosynthesized selenium nanoparticles improve performance, antioxidant status, humoral immunity and dietary and ileal microbial populations of heat-stressed broilers. Journal of Thermal Biology.

[bib0002] Abouelezz F.M.K. (2017). Evaluation of spirulina algae (Spirulina platensis) as a feed supplement for Japanese quail: Nutritional effects on growth performance, egg production, egg quality, blood metabolites, sperm-egg penetration and fertility. Egyptian Poultry Science Journal.

[bib0003] Aguillón-Páez Y.J., Romero L.A., Diaz G.J. (2020). Effect of full-fat sunflower or flaxseed seeds dietary inclusion on performance, egg yolk fatty acid profile and egg quality in laying hens. Animal nutrition (Zhongguo xu mu shou yi xue hui).

[bib0004] Agustini T.W., Suzery M., Sutrisnanto D., Ma’ruf W.F. (2015). Comparative study of bioactive substances extracted from fresh and dried Spirulina sp. Procedia Environmental Sciences.

[bib0005] Akbarian A., Michiels J., Degroote J., Majdeddin M., Golian A., De Smet S. (2016). Association between heat stress and oxidative stress in poultry; mitochondrial dysfunction and dietary interventions with phytochemicals. Journal of Animal Science and Biotechnology.

[bib0006] Akila G., Rajakrishnan V., Viswanathan P., Rajashekaran K.N., Menon V.P. (1998). Effects of curcumin on lipid profile and lipid peroxidation status in experimental hepatic fibrosis. Hepatology Research: The official journal of the Japan Society of Hepatology.

[bib0007] Al-Safi Z.A., Liu H., Carlson N.E., Chosich J., Harris M., Bradford A.P., Polotsky A.J. (2016). Omega-3 fatty acid supplementation lowers serum FSH in normal weight but not obese women. The Journal of Clinical Endocrinology and Metabolism.

[bib0008] Anvar A.A., Nowruzi B. (2021). Bioactive properties of spirulina: A review. Bioactive Materials.

[bib0009] AOAC International (2006). Official methods of analysis of aoac international.

[bib0010] Attia Y.A., Al-Harthi M.A., Al-Sagan A.A., Alqurashi A.D., Korish M.A., Abdulsalam N.M., Bovera F. (2022). Dietary supplementation with different ω-6 to ω-3 fatty acid ratios affects the sustainability of performance, egg quality, fatty acid profile, immunity and egg health indices of laying hens. Agriculture.

[bib0011] Ayerza R., Coates W. (2001). Omega-3 enriched eggs: The influence of dietary α-linolenic fatty acid source on egg production and composition. Canadian Journal of Animal Science.

[bib0012] Bender K., Walsh S., Evans A.C.O., Fair T., Brennan L. (2010). Metabolite concentrations in follicular fluid may explain differences in fertility between heifers and lactating cows. Reproduction (Cambridge, England).

[bib0013] Boiago M.M., Dilkin J.D., Kolm M.A., Barreta M., Souza C.F., Baldissera M.D., Da Silva A.S. (2019). Spirulina platensis in Japanese quail feeding alters fatty acid profiles and improves egg quality: Benefits to consumers. Journal of Food Biochemistry.

[bib0014] Bosch F., Palmeirim M.S., Ali S.M., Ame S.M., Hattendorf J., Keiser J. (2021). Diagnosis of soil-transmitted helminths using the Kato-Katz technique: What is the influence of stirring, storage time and storage temperature on stool sample egg counts. PLoS Neglected Tropical Diseases.

[bib0015] Ceylan N., Ciftçi I., Mizrak C., Kahraman Z., Efil H. (2011). Influence of different dietary oil sources on performance and fatty acid profile of egg yolk in laying hens. Journal of Animal and feed Sciences.

[bib0016] Cherian G. (2008). Egg quality and yolk polyunsaturated fatty acid status in relation to broiler breeder hen age and dietary n-3 oils. Poultry Science.

[bib0017] Chu Q., Yu Y.X., Zhang J.Z., Zhang Y.T., Yu J.P. (2024). Effects of flaxseed oil supplementation on metaphase II oocyte rates in IVF cycles with decreased ovarian reserve: A randomized controlled trial. Frontiers in Endocrinology.

[bib0018] Doğan S.C., Baylan M., Yaman S., Bulancak A. (2018). Effects of dietary licorice root (Glycyrrhiza glabra) supplementation, storage time and temperature on quality of quail eggs. Progress in Nutrition.

[bib0019] Ebeid T., Eid Y., Saleh A., El-Hamid H.Abd (2008). Ovarian follicular development, lipid peroxidation, antioxidative status and immune response in laying hens fed fish oil-supplemented diets to produce n-3-enriched eggs. Anim.

[bib0020] Ebrahimi M., Ghaderi Chaparabad H., Al-Moussawi I.M., Hosseini M.H., Mirghelenj S.A. (2025). Thyme plant powder (Thymus vulgaris) improves the production performance of laying hens by affecting ovarian follicles. Tropical Animal Science Journal.

[bib0021] Ehr I.J., Persia M.E., Bobeck E.A. (2017). Comparative omega-3 fatty acid enrichment of egg yolks from first-cycle laying hens fed flaxseed oil or ground flaxseed. Poultry Science.

[bib0022] Eilati E., Small C.C., McGee S.R., Kurrey N.K., Hales D.B. (2013). Anti-inflammatory effects of fish oil in ovaries of laying hens target prostaglandin pathways. Lipids in Health and Disease.

[bib0023] Elkin R.G., Harvatine K.J. (2023). A review of recent studies on the enrichment of eggs and poultry meat with omega-3 polyunsaturated fatty acids: Novel findings and unanswered questions. Poultry Science.

[bib0024] Faitarone A.B.G., Garcia E.A., Roça R.O., Andrade E.N., Vercese F., Pelícia K. (2016). Yolk color and lipid oxidation of the eggs of commercial white layers fed diets supplemented with vegetable oils. Brazilian Journal of Poultry Science.

[bib0025] Fernandes R., Campos J., Serra M., Fidalgo J., Almeida H., Casas A., Barros A.I. (2023). Exploring the benefits of phycocyanin: From Spirulina cultivation to its widespread applications. Pharmaceuticals.

[bib0026] Fouladi-Nashta A.A., Wonnacott K.E., Gutierrez C.G., Gong J.G., Sinclair K.D., Garnsworthy P.C., Webb R. (2009). Oocyte quality in lactating dairy cows fed on high levels of n-3 and n-6 fatty acids. Reproduction (Cambridge, England).

[bib0027] Fraeye I., Bruneel C., Lemahieu C., Buyse J., Muylaert K., Foubert I. (2012). Dietary enrichment of eggs with omega-3 fatty acids: A review. Food Research International (Ottawa, Ont.).

[bib0028] Gao Z., Zhang J., Li F., Zheng J., Xu G. (2021). Effect of oils in feed on the production performance and egg quality of laying hens. Animals.

[bib0029] Goyens P.L., Spilker M.E., Zock P.L., Katan M.B., Mensink R.P. (2006). Conversion of α-linolenic acid in humans is influenced by the absolute amounts of α-linolenic acid and linoleic acid in the diet and not by their ratio. The American Journal of Clinical Nutrition.

[bib0030] Hajati H., Yousefi Qameshloo A., Hosseini S.A., Ghamsari A.Alizadeh (2024). Bio-functional and nutritional effects of Spirulina algae in commercial laying hens. World’s Poultry Science Journal.

[bib0031] Haugh R.R. (1937). The haugh unit for measuring eggs quality.

[bib0032] Huang J., Hao Q., Wang Q., Wang Y., Wan X., Zhou Y. (2019). Supplementation with green tea extract affects lipid metabolism and egg yolk lipid composition in laying hens. The Journal of Applied Poultry Research.

[bib0033] Huang Q., Liu L., Wu Y., Huang X., Wang G., Song H., Luo P. (2022). Mechanism of differences in characteristics of thick/thin egg whites during storage: Physicochemical, functional and molecular structure characteristics analysis. Food Chemistry.

[bib0034] Irawan A., Ningsih N., Rusli R.K., Suprayogi W.P.S., Akhirini N., Hadi R.F., Jayanegara A. (2022). Supplementary n-3 fatty acids sources on performance and formation of omega-3 in egg of laying hens: A meta-analysis. Poultry Science.

[bib0035] Jin Y.H., Lee K.T., Lee W.I., Han Y.K. (2010). Effects of storage temperature and time on the quality of eggs from laying hens at peak production. Asian-Australasian Journal of Animal Sciences.

[bib0036] Khatibjoo A., Kermanshahi H., Golian A., Zaghari M. (2018). The effect of n-6/n-3 fatty acid ratios on broiler breeder performance, hatchability, fatty acid profile and reproduction. Journal of Animal Physiology and Animal Nutrition.

[bib0037] Kim J., Magnuson A., Tao L., Barcus M., Lei X.G. (2016). Potential of combining flaxseed oil and microalgal biomass in producing eggs-enriched with n-3 fatty acids for meeting human needs. Algal Research.

[bib0038] Kralik G., Kralik Z., Grčević M., Galović O., Hanžek D., Biazik E. (2021). Fatty acid profile of eggs produced by laying hens fed diets containing different shares of fish oil. Poultry Science.

[bib0039] Lee S.H., Kim Y.B., Kim D.H., Lee D.W., Lee H.G., Jha R., Lee K.W. (2021). Dietary soluble flaxseed oils as a source of omega-3 polyunsaturated fatty acids for laying hens. Poultry Science.

[bib0040] Leroy J.L., Sturmey R.G., Van Hoeck V., De Bie J., McKeegan P.J., Bols P.E. (2014). Dietary fat supplementation and the consequences for oocyte and embryo quality: Hype or significant benefit for dairy cow reproduction. Reproduction in Domestic Animals = Zuchthygiene.

[bib0041] Li D., Lv C., Liu L., Xia Y., She X., Guo S., Yang D. (2015). Egg-box structure in cobalt alginate: A new approach to multifunctional hierarchical mesoporous N-doped carbon nanofibers for efficient catalysis and energy storage. ACS Central Science.

[bib0042] Li S., Jing M., Mohamed N., Rey-Dubois C., Zhao S., Aukema H.M., House J.D. (2023). The effect of increasing concentrations of omega-3 fatty acids from either flaxseed oil or preformed docosahexaenoic acid on fatty acid composition, plasma oxylipin, and immune response of laying hens. The Journal of Nutrition.

[bib0043] Mahla A.S., Chaudhari R.K., Verma A.K., Singh A.K., Singh S.K., Singh G., Sarkar M., Dutta N., Kumar H., Krishnaswamy N. (2017). Effect of dietary supplementation of omega-3 polyunsaturated fatty acid (PUFA) rich fish oil on reproductive performance of the goat. TheriogenologyTheriogenology.

[bib0044] Mannion D.T., Furey A., Kilcawley K.N. (2016). Comparison and validation of 2 analytical methods for the determination of free fatty acids in dairy products by gas chromatography with flame ionization detection. Journal of Dairy Science.

[bib0045] Miranda M.S., Cintra R.G., Barros S.B., Mancini-Filho J. (1998). Antioxidant activity of the microalga Spirulina maxima. Brazilian Journal of Medical and Biological Research = Revista Brasileira de Pesquisas Medicas e Biologicas / Sociedade Brasileira de Biofisica ... [et al.].

[bib0046] Mirghelenj S.A., Kianfar R., Janmohammadi H., Taghizadeh A. (2017). Effect of different levels of grape pomace on egg production performance and egg internal quality during different keeping times and temperatures. Animal Production Research.

[bib0047] Mirzaie S., Zirak-Khattab F., Hosseini S.A., Donyaei-Darian H. (2018). Effects of dietary Spirulina on antioxidant status, lipid profile, immune response and performance characteristics of broiler chickens reared under high ambient temperature. Asian-Australasian Journal of Animal Sciences.

[bib0048] Mtaki K., Kyewalyanga M.S., Mtolera M.S. (2020). Assessment of antioxidant contents and free radical-scavenging capacity of chlorella vulgaris cultivated in low cost media. Applied Science.

[bib0049] Musati M., Bertino A., Cannone M.S., Mangano F., Luciano G., Priolo A., Natalello A. (2025). Dietary hazelnut skin prevents lipid oxidation in lamb enriched in omega-3 polyunsaturated fatty acids. Meat Science.

[bib0050] Nampijja Z., Kiggundu M., Kigozi A., Lugya A., Magala H., Ssepuuya G., Mugerwa S. (2023). Optimal substitution of black soldier fly larvae for fish in broiler chicken diets. Scientific African.

[bib0051] Nateghi R., Alizadeh A., Jafari Ahangari Y., Fathi R., Akhlaghi A. (2019). Stimulatory effects of fish oil and vitamin E on ovarian function of laying hen. Italian Journal of Animal Science.

[bib0052] Neijat M., Ojekudo O., House J.D. (2016). Effect of flaxseed oil and microalgae DHA on the production performance, fatty acids and total lipids of egg yolk and plasma in laying hens. Prostaglandins, Leukotrienes, and Essential Fatty Acids.

[bib0053] Omri B., Alloui N., Durazzo A., Lucarini M., Aiello A., Romano R., Abdouli H. (2019). Egg yolk antioxidants profiles: Effect of diet supplementation with linseeds and tomato-red pepper mixture before and after storage. Foods (Basel, Switzerland).

[bib0054] Panaite T.D., Cornescu G.M., Predescu N.C., Cismileanu A., Turcu R.P., Saracila M., Soica C. (2023). Microalgae (Chlorella vulgaris and Spirulina platensis) as a protein alternative and their effects on productive performances, blood parameters, protein digestibility, and nutritional value of laying hens’ egg. Applied Sciences.

[bib0055] Rajesha J., Madhusudhan B., Swamy M.M., Rao R.J., Ravishankar G.A., Rangarao A., Karunakumar M. (2009). Effects of flaxseed and spirulina biomass in layer diet on lipid profile and quality characteristics of egg yolk. Journal of Food Science and Technology-Mysore.

[bib0056] Renema R.A., Robinson F.E., Melnychuk V.L., Hardin R.T., Bagley L.G., Emmerson D.A., Blackman J.R. (1995). The use of feed restriction for improving reproductive traits in male-line large white turkey hens: 2. Ovary morphology and laying traits. Poultry Science.

[bib0057] Rey A.I., Carmona J.M., Daza A., Sanz M., López-Bote C.J. (2010). Quantitative study on fatty acid profile, oxidation and rheological properties of fat and breast muscle from broilers as affected by days of feeding a linoleic acid-enriched diet. Arch. Geflügelkd..

[bib0058] Rouhanipour H., Sharifi S.D., Irajian G.H., Jalal M.P. (2022). The effect of adding L-carnitine to omega-3 fatty acid diets on productive performance, oxidative stability, cholesterol content, and yolk fatty acid profiles in laying hens. Poultry science.

[bib0059] Salahuddin M., Abdel-Wareth A.A., Stamps K.G., Gray C.D., Aviña A.M., Fulzele S., Lohakare J. (2024). Enhancing laying hens’ performance, egg quality, shelf life during storage, and blood biochemistry with Spirulina platensis supplementation. Veterinary Sciences.

[bib0060] Saleh A.A. (2013). Effects of fish oil on the production performances, polyunsaturated fatty acids and cholesterol levels of yolk in hens. Emirates Journal of Food and Agriculture.

[bib0061] Saleh A.A., Ahmed E.A., Ebeid T.A. (2019). The impact of phytoestrogen source supplementation on reproductive performance, plasma profile, yolk fatty acids and antioxidative status in aged laying hens. Reproduction in Domestic Animals = Zuchthygiene.

[bib0062] SAS Institute (2009). STAT user's guide, version 9.2.

[bib0063] Senger P.L. (2005). Pathways to pregnancy and parturition.

[bib0064] Shahryari M., Tabeidian S.A., Shahraki A.D.F., Tabatabaei S.N., Toghyani M., Forouzmand M., Habibian M. (2021). Using soybean acid oil or its calcium salt as the energy source for broiler chickens: Effects on growth performance, carcass traits, intestinal morphology, nutrient digestibility, and immune responses. Animal Feed Science and Technology.

[bib0065] Sherratt S.C., Libby P., Budoff M.J., Bhatt D.L., Mason R.P. (2023). Role of omega-3 fatty acids in cardiovascular disease: The debate continues. Current Atherosclerosis Reports.

[bib0066] Tufarelli V., Baghban-Kanani P., Azimi-Youvalari S., Hosseintabar-Ghasemabad B., Slozhenkina M., Gorlov I., Laudadio V. (2021). Effects of horsetail (Equisetum arvense) and Spirulina platensis dietary supplementation on laying hens productivity and oxidative status. Animals.

[bib0067] Venkatraman J.T., Angkeow P., Satsangi N., Fernandes G. (1998). Effects of dietary n-6 and n-3 lipids on antioxidant defense system in livers of exercised rats. Journal of the American College of Nutrition.

[bib0068] Vui N.V., Oanh D.H., Quyen N.T.K., Linh N.T., Nang K., Phong N.H. (2024). The impact of adding spirulina algae to drinking water on the productivity, egg quality, yolk lipid oxidation, and blood biochemistry of laying hens. Advances in Animal and Veterinary Sciences.

[bib0069] Watanabe K. (2002). Prostaglandin f synthase.

[bib0070] Yoon S., Jeong H., Hong S.J., Jo S.M., Park H., Ban Y., Shin E.C. (2024). Oven-roasting effects the fatty acid composition, antioxidant properties, and oxidative stability of pomegranate (Punica granatum L.) seed oil. Preventive Nutrition and Food Science.

